# Survival dynamical systems: individual-level survival analysis from population-level epidemic models

**DOI:** 10.1098/rsfs.2019.0048

**Published:** 2019-12-13

**Authors:** Wasiur R. KhudaBukhsh, Boseung Choi, Eben Kenah, Grzegorz A. Rempała

**Affiliations:** 1 Mathematical Biosciences Institute, The Ohio State University, Columbus, OH, USA; 2 Division of Economics and Statistics, Department of National Statistics, Korea University Sejong campus, Sejong Special Autonomous City, Republic of Korea; 3 Division of Biostatistics, College of Public Health, The Ohio State University, Columbus, OH, USA; 4 Division of Biostatistics, College of Public Health and Mathematical Biosciences Institute, The Ohio State University, Columbus, OH, USA

**Keywords:** epidemic models, survival analysis, stochastic processes, dynamical systems, multiscale models

## Abstract

In this paper, we show that solutions to ordinary differential equations describing the large-population limits of Markovian stochastic epidemic models can be interpreted as survival or cumulative hazard functions when analysing data on individuals sampled from the population. We refer to the individual-level survival and hazard functions derived from population-level equations as a survival dynamical system (SDS). To illustrate how population-level dynamics imply probability laws for individual-level infection and recovery times that can be used for statistical inference, we show numerical examples based on synthetic data. In these examples, we show that an SDS analysis compares favourably with a complete-data maximum-likelihood analysis. Finally, we use the SDS approach to analyse data from a 2009 influenza A(H1N1) outbreak at Washington State University.

## 1. Introduction

Despite their ubiquity in modern epidemiology, mathematical models of epidemics suffer many theoretical and practical drawbacks. Due to the need for mathematical tractability, such models often ignore important characteristics of disease transmission patterns and the underlying populations. This often leads to poor predictions. During the SARS epidemic of 2002–2003, the number of cases in China was predicted to reach 30 000 during the first four months of the epidemic. In fact, there were fewer than 800 cases reported during that time [1]. A more recent example is the Centers for Disease Control and Prevention (CDC) prediction of the 1 400 000 cases of Ebola in West Africa during 2013–2016 outbreak [2,3]. Although the CDC team did indicate that their prediction was the ‘worst-case scenario’, the inaccuracy of this upper bound prediction has highlighted the need for better mathematical models of epidemics and their control.

A typical challenge in the problem of epidemic control is how to relate the global, population-level dynamics of infection transmission to local, individual-level intervention (e.g. vaccination). This dichotomy is reflected in two distinct approaches to modelling epidemiological processes. Agent-based models capture individual-level histories of infection and removal. By contrast, ecological models look at the population at an aggregate level, keeping track of summary statistics such as the counts of susceptible, infected and recovered/removed individuals. Although both agent-based and ecological models are routinely used in practice and in the literature, the two scales of analysis are almost always considered separately [4].

The Kermack–McKendrick model [5] is the most fundamental example of an ecological model. It assumes the population is segregated into susceptible (S), infected (I) and recovered/removed (R) compartments. The time evolutions of the population proportions in compartments (denoted by *S*_*t*_, *I*_*t*_ and *R*_*t*_) are described by the following well-known system of ordinary differential equations (ODEs):

1.1 $$\begin{array}{rl} {\dot{S}}_{t} & =-\beta S_{t}I_{t},\\ {\dot{I}}_{t} & =\beta S_{t}I_{t}-\gamma I_{t}\\ \;\mathrm{and}\;{\dot{R}}_{t} & =\gamma I_{t}. \end{array}\}$$

**Figure 1.**
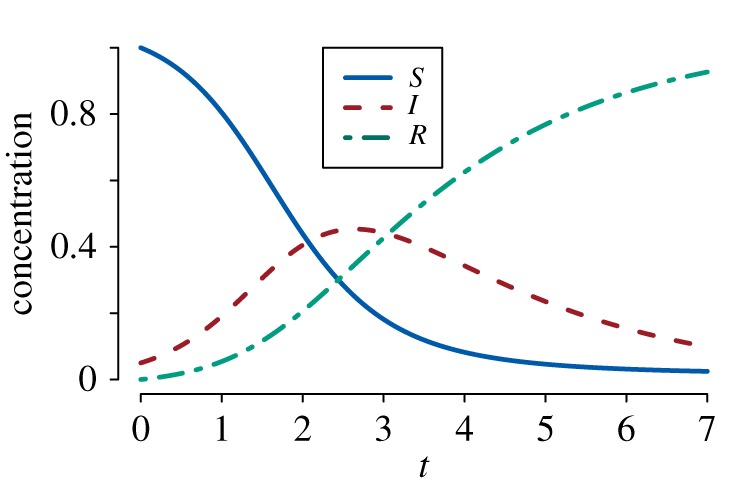
SDS interpretation of the SIR curves. The *S*_*t*_ curve is the survival function for time to infection: $S_{t}=P(T_{I}>t)$ where *T*_*I*_ is the time at which an individual moves from the susceptible to the infected compartment. The *R*_*t*_ curve, upon multiplication with $R_{0}$, gives the corresponding cumulative hazard. Finally, the convolution of the infection time *T*_*I*_ and the infectious time *T*_*R*_ (time spent in the infected compartment) is given by the *I*_*t*_ curve, after adjustment for the initial infecteds. Parameter values: *β* = 2, *γ* = 0.5 with initial condition *S*_0_ = 1, *I*_0_ = 0.05 and *R*_0_ = 0. (Online version in colour.)

Here, *β* and *γ* are the infection and recovery rates, respectively. Solutions to equation (1.1) are often called the susceptible–infected–recovered (SIR) curves (figure 1). The law of mass action has been implicitly assumed, so any infectious individual can infect any susceptible individual. The ODEs model in equation (1.1) averages out individual dynamics, so it does not capture the stochastic fluctuation of epidemic processes in real life. In particular, the practical problems of applying equation (1.1) to data are:

1. **Population size.** Since the quantities in the SIR equations are proportions, it is not immediately clear how to apply them to real epidemics, which occur in *finite* susceptible populations. Moreover, the size of the population is often unknown.
2. **Likelihood.** Since the SIR equations are deterministic, we cannot write a likelihood for epidemic data without further, often *ad hoc*, statistical assumptions about the form of the likelihood function.
3. **Aggregation over individuals.** The SIR model represents the mean-field equations for (scaled) population counts, aggregating out individual characteristics.
4. **Aggregation over time.** The real data are typically aggregated not just over the population but also over observed time periods, leading to interval censoring^1^ that cannot be easily incorporated into the SIR equations.

In this paper, we show that simple algebraic manipulation of the SIR equation (1.1) uncovers a precise probability law for the individual transitions between compartments. We refer to this interpretation of the solutions of equation (1.1) as a survival dynamical system (SDS). This new interpretation allows us to apply tools from survival analysis to population-level epidemic data. It directly addresses the first two problems listed above, and it lays a theoretical foundation for addressing the latter two problems. We focus on Markovian mass-action SIR models in this paper, but the SDS approach generalizes to non-Markov and network-based epidemic models.

The rest of the paper is structured as follows. First, we briefly review the relevant background on mathematical modelling in epidemiological literature. In §2, we make the SDS interpretation of the SIR equation (1.1) precise. In §3, we show how this approach can be used for statistical inference and compare the performance of estimators based on SDS likelihoods to those based on standard complete-data likelihoods. In §4, we use an SDS likelihood to analyse 2009 influenza A(H1N1) outbreak data from Washington State University. Finally, we conclude the paper with a brief discussion in §5. Additional mathematical preliminaries, statistical inference results and other material are provided in the appendices. A list of symbols used in the paper is provided in table 1.

**Table 1.** List of symbols.

symbol	meaning
*β*	infection rate
*γ*	recovery rate
*ρ*	fraction of initially infected population
*τ*	final size of the epidemic
*T*	end of observation period
$R_{0}$	basic reproduction number
*S*_*i*_(*t*), *I*_*i*_(*t*), *T*_*i*_(*t*)	indicator functions taking value 1 if at time *t*, *i* is, respectively, susceptible, infected or removed and 0 otherwise
*S*(*t*), *I*(*t*), *R*(*t*)	numbers of susceptible, infected and recovered individuals at time *t*
*T*_*i*,*I*_, *T*_*i*,*R*_	the times of infection and recovery of *i*
*T*_*I*_, *T*_*R*_	the times of infection and recovery of a randomly chosen individual
*W*	the infectious period, i.e. *W* := *T*_*R*_ − *T*_*I*_
*f*_*τ*_	the density of *T*_*I*_ conditional on *T*_*I*_ < ∞
*g*_*τ*_	the density of *T*_*R*_

### 1.1. Individual level: agent-based susceptible–infected–recovered model

Suppose we have *n* susceptible and *m* infectious individuals initially. Infectious individuals infect susceptible individuals, who change state from susceptible to infected. Infected individuals recover after an exponential infectious period. All infectious contacts and recoveries are assumed independent of each other. For the *i*-th individual, define the process *S*_*i*_ such that *S*_*i*_(*t*) = 1 if he or she is in the susceptible compartment at time *t* and *S*_*i*_(*t*) = 0 otherwise. Similarly, define the processes *I*_*i*_ for the infected compartment and *R*_*i*_ for the recovered compartment. Naturally, *S*_*i*_(*t*) + *I*_*i*_(*t*) + *R*_*i*_(*t*) = 1. For time *T* ∈ (0, ∞), we assume that the process {(*S*_*i*_(*t*), *I*_*i*_(*t*), *R*_*i*_(*t*))}_*i*=1, …,*n*+*m*; *t*∈[0,*T*]_ is a continuous-time Markov chain (CTMC). For notational convenience, we have labelled the initial susceptible individuals 1, 2, …, *n* and the initial infectious individuals *n* + 1, *n* + 2, …, *n* + *m*. Then the random time change representation of a CTMC (see [6, ch. 6, pp. 326–328], [7, eqn 5.2, ch. 5, p. 41] and [8, eqn 1.8, ch. 1, p. 11]) allows us to write, for each *i* ∈ {1, …, *n* + *m*},

1.2 $$\begin{array}{rl} & S_{i}(t)=S_{i}(0)-Y_{i}(\int_{0}^{t}\frac{\beta }{n}S_{i}(s)\sum_{\;j=1}^{n+m}I_{\;j}(s)\;\text{d}s),\\ & I_{i}(t)=I_{i}(0)+Y_{i}(\int_{0}^{t}\frac{\beta }{n}S_{i}(s)\sum_{\;j=1}^{n+m}I_{\;j}(s)\;\text{d}s)-Z_{i}(\int_{0}^{t}\gamma I_{i}(s)\;\text{d}s)\\ \text{and}\; & R_{i}(t)=Z_{i}(\int_{0}^{t}\gamma I_{i}(s)\;\text{d}s), \end{array}\}$$

where *Y*_1_, *Y*_2_, …, *Y*_*n*+*m*_ and *Z*_1_, *Z*_2_, …, *Z*_*n*+*m*_ are independent unit-rate Poisson processes. Models of this form are often called agent-based models in the literature [9,10].

An intuitive explanation behind the random time change represetation in equation (1.2) is as follows: consider individual *i* who is initially susceptible. He or she will change status from susceptible to infected as soon as one of the infected individuals make an infectious contact. Because infected individuals make infectious contacts independently, the amount of time the *i*-th individual will remain susceptible has an exponential distribution with rate $n^{-1}\beta \sum_{\;j=1}^{n+m}I_{\;j}$. Once infected, he/she cannot be infected again. Therefore, the jump of the local process *S*_*i*_ from 1 to 0 can be equivalently described by the jump of the process $Y_{i}(\int_{0}^{t}n^{-1}\beta S_{i}(s)\sum_{\;j=1}^{n+m}I_{\;j}(s)\;\text{d}s)$, where *Y*_*i*_ is a unit-rate Poisson process. Note that when the local process *S*_*i*_ jumps from 1 to 0, the process *I*_*i*_ also jumps from 0 to 1. When *i* is in infected status, he/she will recover after an exponentially distributed amount of time with rate *γ*. Therefore, the jump of the local process *I*_*i*_ from 1 to 0 can be equivalently described by the jump of $Z_{i}(\int_{0}^{t}\gamma I_{i}(s)\;\text{d}s)$, where *Z*_*i*_ is a unit-rate Poisson process. Similar arguments give the equation for the local process *R*_*i*_. The random time change representation in equation (1.2) for the entire ensemble {(*S*_*i*_(*t*), *I*_*i*_(*t*), *R*_*i*_(*t*))}_*i*=1, …,*n*+*m*; *t*∈[0,*T*]_ follows from these considerations.

An equivalent construction of the agent-based model in equation (1.2) was proposed by Sellke [11]. Let *T*_*i*,*I*_ denote the amount of time *i* remains susceptible, provided he or she was susceptible initially. Given the history of the infection process $I(s)=\sum_{\;j=1}^{m+n}I_{\;j}(s)$ up to time *t*, the conditional probability that individual *i* remains susceptible until time *t* is given by

1.3 $$P(T_{i,I}>t|{\left(I(s)\right)}_{s\in [0,t]})=\exp\left(-\frac{\beta }{n}\int_{0}^{t}I(s)\;\text{d}s\right).$$

Therefore, to each susceptible individual *i*, we can assign an independent Exponential(1) random variable *Q*_*i*_ and change his/her status from susceptible to infected when

$$Q_{i}>\Lambda (t):=\frac{\beta }{n}\int_{0}^{t}I(s)\;\text{d}s.$$

Once a susceptible individual gets infected, he or she recovers after an infectious period that follows an exponential distribution with rate *γ*. If we denote the recovery time of the *i*-th individual by *T*_*i*,*R*_, it follows immediately from equation (1.2) that *T*_*i*,*R*_ − *T*_*i*,*I*_ and *T*_*i*,*I*_ are independent and *T*_*i*,*R*_ − *T*_*i*,*I*_ has an exponential distribution with rate *γ*. Symbolically,

1.4 $$T_{i,R}-T_{i,I}⊥T_{i,I}\;\text{and}\;T_{i,R}-T_{i,I}∼E\mathrm{XPONENTIAL}(\gamma ).$$

The fate of an individual is entirely described by the statistical distributions given in equations (1.3) and (1.4). The Sellke construction can also be derived using a statistical representation of agent-based models under the law of mass action based on contact intervals [12,13]. In this case, the contact interval distribution is Exponential(*β*).

These considerations lead to algorithm 1.1 for simulating the process in equation (1.2), which is known as the *Sellke construction* [7,14,15]. It can be easily verified that algorithm 1.1 is equivalent to simulating the system in equation (1.2).


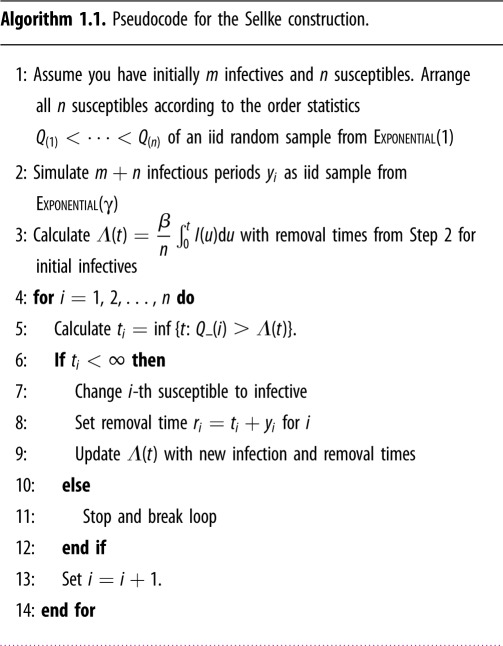


### 1.2. Population level: ecological susceptible–infected–recovered model

The simplest way to derive an ecological model from the agent-based model in equation (1.2) is via lumping or aggregation of states. When the aggregation of states is *strongly lumpable* [16,17] (also see appendix A), the resulting aggregated process remains Markovian for any choice of the initial distribution. For the SIR process, let X:={S,I,R} denote the state space of each individual. Then, $X^{n+m}$ is the state space of the ensemble of individual-based *S*_*i*_, *I*_*i*_, *R*_*i*_ processes. Define the macro-level processes

1.5 $$S(t)=\sum_{i=1}^{n+m}S_{i}(t),I(t)=\sum_{i=1}^{n+m}I_{i}(t)\;\text{and}\;R(t)=\sum_{i=1}^{n+m}R_{i}(t),$$

which keep track of the total counts of susceptible, infected and recovered individuals. Let $L:=\left(\begin{array}{c} n+m+2\\ 2 \end{array}\right)$. Partition $X^{n+m}$ into $X_{1},X_{2},\ldots ,X_{L}$ such that any two states in each $X_{l}$ produce the same counts for *S*(*t*), *I*(*t*), *R*(*t*), for *l* = 1, 2, …, *L*. It is easy to see that the Markov chain described in equation (1.2) is (strongly) lumpable with respect to the partition $\left\{X_{1},X_{2},\ldots ,X_{L}\right\}$ (see [10,17,18]). That is, the lumped process (*S*, *I*, *R*) is also Markovian for any choice of the initial distribution. Therefore, we can write

1.6 $$\begin{array}{rl} & S(t)=S(0)-Y(\int_{0}^{t}\frac{\beta }{n}S(s)I(s)\;\text{d}s),\\ & I(t)=I(0)+Y(\int_{0}^{t}\frac{\beta }{n}S(s)I(s)\;\text{d}s)-Z(\int_{0}^{t}\gamma I(s)\;\text{d}s)\\ \;\text{and}\; & R(t)=Z(\int_{0}^{t}\gamma I(s)\;\text{d}s), \end{array}\}$$

where *Y* and *Z* are independent unit-rate Poisson processes. This system can be simulated using the Doob–Gillespie algorithm (see algorithm B.1 in appendix B).

This ecological model is convenient in that it is amenable to asymptotic analysis. Indeed, for very large populations, we can approximate the scaled stochastic SIR dynamics by a system of ODEs [19,20]. This is sometimes called *mean-field* or *fluid limit* of the Markov jump process. For our SIR system in equation (1.6), the scaled process (*S*_*n*_, *I*_*n*_, *R*_*n*_) := (*S*/*n*, *I*/*n*, *R*/*n*) satisfies

1.7 $$\begin{array}{rl} & S_{n}(t)=S_{n}(0)-\frac{1}{n}Y(n\int_{0}^{t}\beta S_{n}(s)I_{n}(s)\;\text{d}s),\\ & I_{n}(t)=I_{n}(0)+\frac{1}{n}Y(n\int_{0}^{t}\beta S_{n}(s)I_{n}(s)\;\text{d}s)-\frac{1}{n}Z(n\int_{0}^{t}\gamma I_{n}(s)\;\text{d}s)\\ \;\text{and}\; & R_{n}(t)=\frac{1}{n}Z(n\int_{0}^{t}\gamma I_{n}(s)\;\text{d}s). \end{array}\}$$

By virtue of the Poisson law of large numbers (LLN) [6], which asserts that *n*^−1^ *V*(*nt*) ≈ *t* for a unit-rate Poisson process *V* when *n* is large, the processes in equation (1.7) converge to the solution of the following system of ODEs as *n* → ∞ and *m*/*n* → *ρ* ∈ (0, 1):

1.8 $$\dot{s_{t}}=-\beta s_{t}\iota_{t},\;\dot{\iota_{t}}=\beta s_{t}\iota_{t}-\gamma \iota_{t}\;\text{and}\;\dot{r_{t}}=\gamma \iota_{t}.$$

These are identical to the Kermack–McKendrick ODEs in equation (1.1). The introduction of *ρ* is convenient because it sets *s*_0_ = 1, $\iota_{0}=\rho$ and *r*_0_ = 0. The rate of convergence to this LLN ODEs limit can be computed using sample path large deviations principle (LDP) of the Markov process in equation (1.7). Standard tools from [21–23] as well as related results from [24–26] can be borrowed for this purpose.

## 2. Survival dynamical systems

The ODEs in equation (1.8) that describe the large-population limit of the ecological SIR model can be given an agent-based probabilistic interpretation. It is convenient to rewrite equation (1.8) as follows:

2.1 $$\begin{array}{rl} & s_{t}=\exp\left(-\beta \int_{0}^{t}\iota_{u}\;\text{d}u\right)=\exp(-R_{0}r_{t}),\\ & \iota_{t}=\rho {\text{e}}^{-\gamma t}-\int_{0}^{t}\dot{s_{u}}{\text{e}}^{-\gamma (t-u)}\;\text{d}u\\ \text{and}\;\;\; & r_{t}=\gamma \int_{0}^{t}\iota_{u}\;\text{d}u, \end{array}\}$$

where $R_{0}=\beta /\gamma$ is the basic reproduction number. Here, the first two equations are obtained by partially solving the ODEs system using the integrating factor (first equation) and variation of parameter (second equation) methods.

In the limit of a large population, the time of infection *T*_*I*_ of a randomly chosen susceptible individual has the survival function

2.2 $$P(T_{I}>t)=s_{t}=\exp(-R_{0}r_{t}).$$

This is a direct analogue of equation (1.3) where the stochastic quantity $n^{-1}\int_{0}^{t}I(u)\;\text{d}u$ is replaced by its deterministic limit $\int_{0}^{t}\iota_{u}\;\text{d}u$ from equation (2.1). Similarly, $R_{0}r_{t}=\beta \int_{0}^{t}\iota_{s}\text{d}s$ may be thought of as the cumulative hazard and $\beta \iota_{t}$ as the hazard function of the random variable *T*_*I*_. This hazard is sometimes called the *force of infection*. In the limit of large *n*, the units become independent due to the phenomenon known as *mean-field independence* or *propagation of chaos* [27–29].

Because *T*_*I*_ is an improper random variable, its survival, cumulative hazard and hazard functions are also improper. The probability that *T*_*I*_ = ∞ equals *s*_∞_, which is the limiting proportion of individuals who remain susceptible. Setting *s*_∞_ = 1 − *τ* and *τ* = *r*_∞_ − *ρ* where *r*_∞_ is the limiting proportion of recovered individuals, we see that *τ* must satisfy the deterministic final size equation

2.3 $$1-\tau =\exp(-R_{0}(\tau +\rho )).$$

The final size equation is a contraction map, so it is amenable to numerically efficient fixed-point iteration schemes. Because 0 ≤ *τ* < 1, we may interpret *τ* as the probability that *T*_*I*_ < ∞. Given that *T*_*I*_ < ∞, its conditional survival function is

2.4 $${\tilde{s}}_{t}=\frac{s_{t}-(1-\tau )}{\tau }$$

and its probability density is

2.5 $$f_{\tau }(t)=-\frac{{\dot{s}}_{t}}{\tau }.$$

Let *T*_*R*_ be the time of removal of an infected individual who is infected at time *T*_*I*_ (with *T*_*I*_ < *T*_*R*_), and let

2.6 $${\tilde{\iota }}_{t}=\iota_{t}-\rho \exp(-\gamma t)\;$$

be the infected proportion of the population excluding the remaining initial infecteds. From equations (2.1) and (2.5), we obtain

2.7 $$\frac{\gamma {\tilde{\iota }}_{t}}{\tau }=\int_{0}^{t}f_{\tau }(u)\;\gamma {\text{e}}^{-\gamma (t-u)}\;\text{d}u.$$

Because $f_{\tau }(u)$ is a density function, the right-hand side above is a convolution of the conditional density *f*_*τ*_ of *T*_*I*_ and the (exponential) density of *T*_*R*_ − *T*_*I*_, the infectious period. It follows that the right-hand side quantity

**Figure 2.**
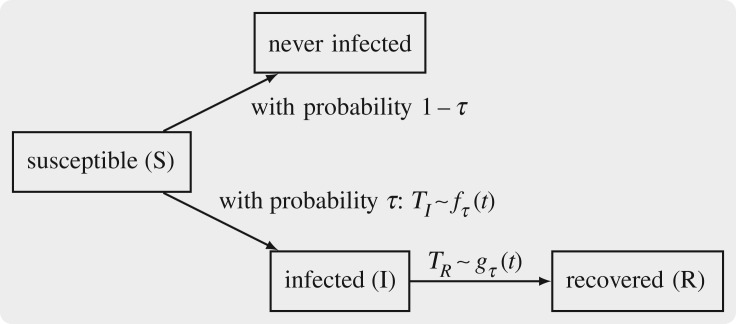
SDS derived from SIR equation (1.1). To each individual, we assign random variables *T*_*I*_ and *T*_*R*_ specifying his/her infection and recovery times, respectively. The laws of *T*_*I*_ and *T*_*R*_ are given by equations (2.5) and (2.8).


2.8 $$g_{\tau }(t)=\frac{\gamma {\tilde{\iota }}_{t}}{\tau }\;$$

is itself a density of the variable *T*_*R*_, which is the sum of the independent random variables *T*_*I*_ and *T*_*R*_ − *T*_*I*_. Note the analogy of this result with equation (1.4). Let us denote the infectious period by the random variable *W* := *T*_*R*_ − *T*_*I*_. These considerations give us algorithm 2.1 for simulating individual histories in the SIR model. See figure 2 for a pictorial representation of the idea.

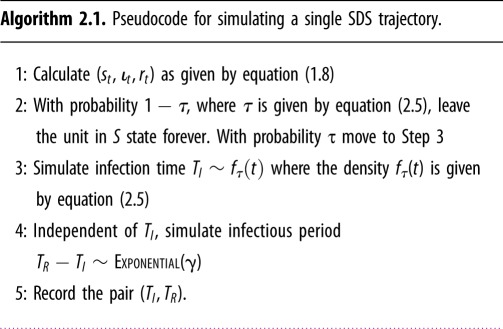


Analysing timepoints (*T*_*I*_, *T*_*R*_) according to algorithm 2.1 addresses all four issues of macro SIR model in equation (1.1) described in §1. Algorithm 2.1 no longer requires the population size (problem 1). Generation of individual trajectories according to algorithm 2.1 allows us to specify a likelihood function (problem 2), account for differences in individual characteristics (problem 3), and overcome issues with censoring or interval-based data (problem 4). Algorithm 2.1 brings us back from ecological to agent-based models and completes a conceptual ‘micro-macro-micro’ loop. The SDS interpretation has similarities with *symbolic dynamical systems* [30–32].

## 3. Parameter estimation

Under the stochastic agent-based SIR model equation (1.2) or its aggregated ecological version in equation (1.6), the vector of parameters of interest is *θ* = (*β*, *γ*, *ρ*) with *m* = *I*(0) = *ρn*. The parameter *τ* is expressible in terms of *θ* via equation (2.3). The size of the initial susceptible population (*n*) is usually unknown and may be considered a nuisance parameter. The estimation of this nuisance parameter is often problematic, and popular methods such as profile likelihoods do not always yield good estimates. In order to address this problem, we propose a likelihood based on the SDS interpretation of the SIR model in equation (1.1) that does not require *n* (although *n* still may be estimated, see algorithm 4.1 in §4). Before going into the details of SDS likelihood, we describe the exact likelihood based on the Doob–Gillespie algorithm (see algorithm B.1 in appendix B). To emphasize the utility of the SDS likelihood, we compare its performance to an exact likelihood that is given the correct value of *n*.

### 3.1. Exact (Doob–Gillespie) likelihood

Assume we observe a total of *z* = *z*_*I*_ + *z*_*R*_ events ${\left(k_{i},t_{i}\right)}_{i=0}^{z}$ at times 0 < *t*_1_ < · · · < *t*_*z*_ = *T* where $k_{i}\in \{\text{I},\text{R}\}$ denotes the type of event. Of these events, *z*_*I*_ are infections and *z*_*R*_ are removals. Put *X*(*t*) = (*S*(*t*), *I*(*t*), *R*(*t*)). Then, following algorithm B.1, the exact log-likelihood for *θ* is

3.1 $$\begin{array}{rl} \ell_{1}(\theta |X{\left(t\right)}_{t\in [0,T]}) & =\sum_{i=1}^{z}\log(\lambda_{k_{i}}(X(t_{i})))-\int_{0}^{T}[\lambda_{I}(X(t))+\lambda_{R}(X(t))]\;\text{d}t\\ & =z_{I}\log(\beta )+z_{R}\log(\gamma )+\sum_{i:k_{i}=I}\log(S(t_{i})/n)\\ & \;+\sum_{i=1}^{z}\log(I(t_{i}))-\int_{0}^{T}\frac{\beta }{n}S(t)I(t)\;\text{d}t-\int_{0}^{T}\gamma I(t)\;\text{d}t, \end{array}$$

where the last two integrals may be also written as finite sums. It is important to note that the above likelihood is conditional on the initial value *X*(0) = (*n*, *ρn*, 0), which we assume to be known. From equation (3.1), the maximum-likelihood estimate (MLE) for *β* and *γ* can be derived as

3.2 $$\hat{\beta }=\frac{nz_{I}}{\int_{0}^{T}S(t)I(t)\;\text{d}t}\;\text{and}\;\hat{\gamma }=\frac{z_{R}}{\int_{0}^{T}I(t)\;\text{d}t}.$$

Because we know the population size *n* and the trajectory *X*(*t*)_*t*∈[0,*T*]_ when using the exact likelihood, the parameter *ρ* = *n*^−1^ *I*(0) is also known exactly.

### 3.2. Survival dynamical system likelihood

Following the discussion in §2, an approximation of the exact likelihood function ℓ_1_(*θ*) in equation (3.1) can be obtained from equation (1.3) by replacing the process *n*^−1^ *I*(*u*) with its asymptotic limit ι (as *n* → ∞) and considering the individual trajectories as independent. Since we let *n* → ∞, the exact value of the initial size of the susceptible population is no longer needed.

Assume we randomly sample *N* + *M* individuals of whom *N* are initially susceptible and *M* initially infected. We observe these *N* + *M* individuals up to the cut-off time *T* and record their infection or recovery times. Suppose *K* out of the *N* initially susceptible individuals get infected at times *t*_1_, *t*_2_, …, *t*_*K*_ and *L* of them recover by time *T*. Pair each infection time *t*_*i*_ with the corresponding duration of infectious period *w*_*i*_ if the individual recovers by time *T*. If the individual does not recover by time *T*, pair *t*_*i*_ with the censored recovery period *w*_*i*_ = *T* − *t*_*i*_. Among the *M* initially infected individuals, suppose $\tilde{L}$ individuals recover by the cut-off *T* at times $\epsilon_{1},\epsilon_{2},\ldots ,\epsilon_{\tilde{L}}$. Then, following algorithm 2.1, we have the following SDS likelihood:

3.3 $$\begin{array}{rl} \ell_{2}(\theta |{\left\{t_{i},w_{i}\right\}}_{i=1}^{K},{\left\{\epsilon_{\;j}\right\}}_{\;j=1}^{\tilde{L}}) & =(N-K)\log(s_{T})+\sum_{i=1}^{K}\log(\tau f_{\tau }(t_{i}))\\ & \;+(L+\tilde{L})\log(\gamma )-\gamma \left(\sum_{i=1}^{K}w_{i}+\sum_{\;j=1}^{\tilde{L}}\epsilon_{\;j}+(M-\tilde{L})T\right), \end{array}$$

where, as described in §2,

$$f_{\tau }(t)=\beta \tau^{-1}\iota_{t}\exp(-R_{0}t_{t}),\;s_{t}=\exp(-R_{0}r_{t}),$$

and *τ* = *r*_∞_ − *ρ* satisfies equation (2.3). In the next section, we evaluate the performance of the SDS likelihood from equation (3.3) in MLE and Markov chain Monte Carlo (MCMC) implementations.

### 3.3. Bayesian estimation using Markov chain Monte Carlo

In order to construct a posterior distribution for *θ*, we assign gamma priors to the parameters *β*, *γ* and *ρ*:

3.4 $$\begin{array}{rl} \beta & ∼G\mathrm{AMMA}\;(a_{\beta },b_{\beta }),\\ \gamma & ∼G\mathrm{AMMA}\;(a_{\gamma },b_{\gamma })\\ \text{and}\;\rho & ∼G\mathrm{AMMA}\;(a_{\rho },b_{\rho }). \end{array}\}$$

The positive quantities *a*_*β*_, *b*_*β*_, *a*_*γ*_, *b*_*γ*_, *a*_*ρ*_ and *b*_*ρ*_ are appropriately chosen hyper-parameters. The posterior distribution of *θ* is obtained by Bayes’ rule: it is proportional to the product of the likelihood function given in equation (3.3) and the three priors above.

Unfortunately, the posterior distribution of the SDS likelihood cannot be written in closed form. Even if a conditional posterior distribution is obtained, any closed-form expression for the probability density function would require solutions *s*_*t*_, $\iota_{t}$, *r*_*t*_ to equation (2.1), which are themselves functions of *θ*. Thus, we cannot employ a generic Gibbs sampler method [33,34], and we need a more efficient updating algorithm than the standard Metropolis–Hastings algorithm. Here, we adopt the robust adaptive metropolis (RAM) algorithm [35,36], which adapts the tuning constant and the variance–covariance matrix of the proposal distribution to maintain a consistent acceptance ratio in the Metropolis steps, which helps achieve good mixing of the chain. The variance–covariance matrix is updated during the MCMC iterations. In algorithm B.2, in appendix B, we provide pseudocode for implementing an MCMC procedure for drawing posterior samples using RAM.

### 3.4. Simulation study

The SDS likelihood presented in the previous section has several theoretical advantages. Two of the main advantages are: (a) it does not require knowledge of the number of initially susceptible individuals *n* and (b) it works with partial data in that it requires trajectories of *only* a randomly chosen sample of individuals. Nevertheless, the SDS likelihood is based on an LLN approximation of a large population, so it is important to evaluate the accuracy of this approximation. In this section, we compare the accuracy of the inference based on the SDS likelihood (without *n*) to that of the exact likelihood (with *n*). Though the comparison is deliberately unfair in that exact value of *n* and full data trajectories are supplied only to the exact likelihood, our objective is to see how much worse the inferences from the SDS likelihood are due to the approximation error as well as lack of *n* and full data trajectories. The data used for parameter inference are generated according to algorithm 1.1.

We compare three different inference methods:

1. **Method 1** uses the Doob–Gillespie likelihood given in equation (3.1) and calculates MLE according to equation (3.2).
2. **Method 2** also uses the Doob–Gillespie likelihood given in equation (3.1), but implements an MCMC scheme with the priors listed in equation (3.4) to infer *θ*. Because of conjugacy of the gamma priors, the posteriors are also gamma distributions [33]. In particular, they are given by
  $$\begin{array}{rl} & \beta |{\left(X(t\right)}_{t\in [0,T]})∼G\mathrm{AMMA}\left(nz_{I}+a_{\beta },\int_{0}^{T}S(t)I(t)\;\text{d}t+b_{\beta }\right)\\ & \text{and}\;\gamma |{\left(X(t\right)}_{t\in [0,T]})∼G\mathrm{AMMA}\left(z_{R}+a_{\gamma },\int_{0}^{T}I(t)\;\text{d}t+b_{\gamma }\right). \end{array}$$
3. **Method 3** uses the SDS likelihood given in equation (3.3) and follows the MCMC procedure described in algorithm B.2.

For all MCMC-based methods, we constrain the proposed values of *ρ* in the MCMC iteration steps so that *ρ* remains within (0, 1) and satisfies equation (2.3). We have a total of 18 simulation scenarios based on combinations of the following:

— Three values of *θ* = (*β*, *γ*, *ρ*): *θ*_1_ = (2.0, 0.5, 0.05), *θ*_2_ = (2.0, 1.0, 0.05) and *θ*_3_ = (1.5, 1.0, 0.05) yielding $R_{0}$ equal to 4, 2 and 1.5, respectively.
— Two cut-off times *T*. Since the epidemic curve sees an exponential growth phase near the beginning, one often runs into problems such as overestimation of the size of the outbreak if inference is done using data collected when the epidemic is at or just before its peak. In order to see the impact of the censoring time *T*, we choose two cut-off times. One cut-off time is chosen around the half-time of the epidemic duration (near the peak of the infection process) and another one towards the end. The chosen values of *T* in our simulation set-up are 3 and 9 for *θ*_1_, 3 and 7 for *θ*_2_, and 3 and 6 for *θ*_3_. See figure 8 for the SIR curves for different parameter values and cut-off times. The vertical line in each plot represents the cut-off time.
— Three values of the size of the susceptible population *n*: 10^2^, 10^3^ and 10^4^.

For each of the 18 scenarios, we generate 100 sets of synthetic epidemic data using algorithm 1.1. Each generated dataset has *n* + *n* × *ρ* rows (one for each individual in the epidemic) and two columns (one for *T*_*I*_ and one for *T*_*R*_). To ensure the prior distributions in our Bayesian inference are uninformative, we set *a*_*i*_ = *i* × 0.01 and *b*_*i*_ = 0.01 for *i* = *β*, *γ* and *ρ*. For Method 2, we generate 1000 samples without any burn-in phase or thinning because Monte Carlo simulations are sufficient. For Method 3, we iterated the MCMC procedures 11 000 times. The first 1000 iterations are removed as burn-in. After burn-in, every 10th iteration is stored as a posterior sample. In total, 1000 posterior samples are used for estimation. For the Bayesian methods (i.e. Method 2 and Method 3), we estimate the parameters *β*, *γ* and *ρ* by taking the means of 1000 posterior samples.

**Figure 3.**
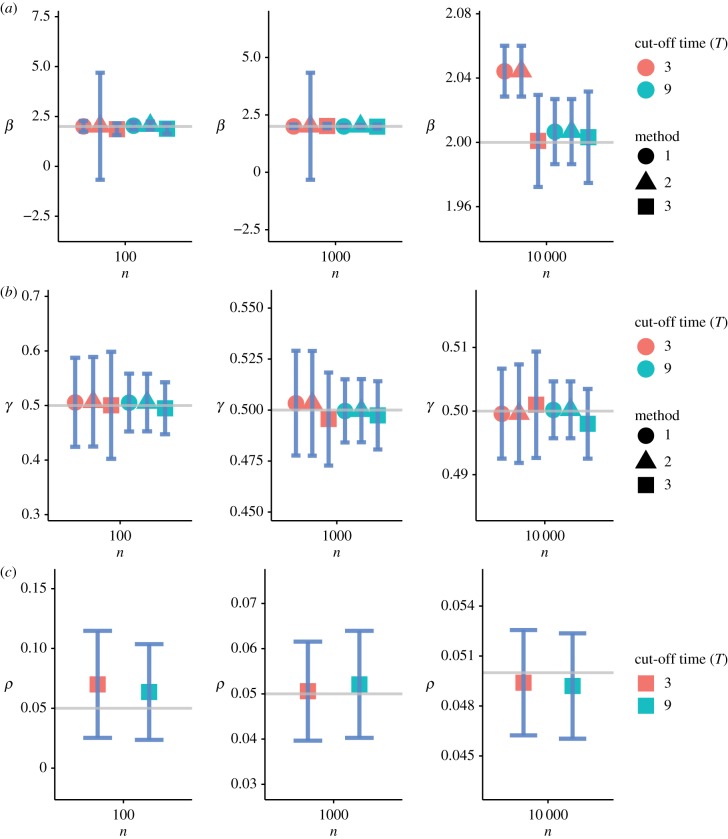
Inference under the parameter setting *θ*_1_. (*a*–*c*) The parameters *β*, *γ* and *ρ*, respectively. The solid grey lines correspond to the true parameter values. The error bars correspond to ±1.96 s.d. of each estimate. The parameter *ρ* is estimated by Method 3 only. (Online version in colour.)

**Figure 4.**
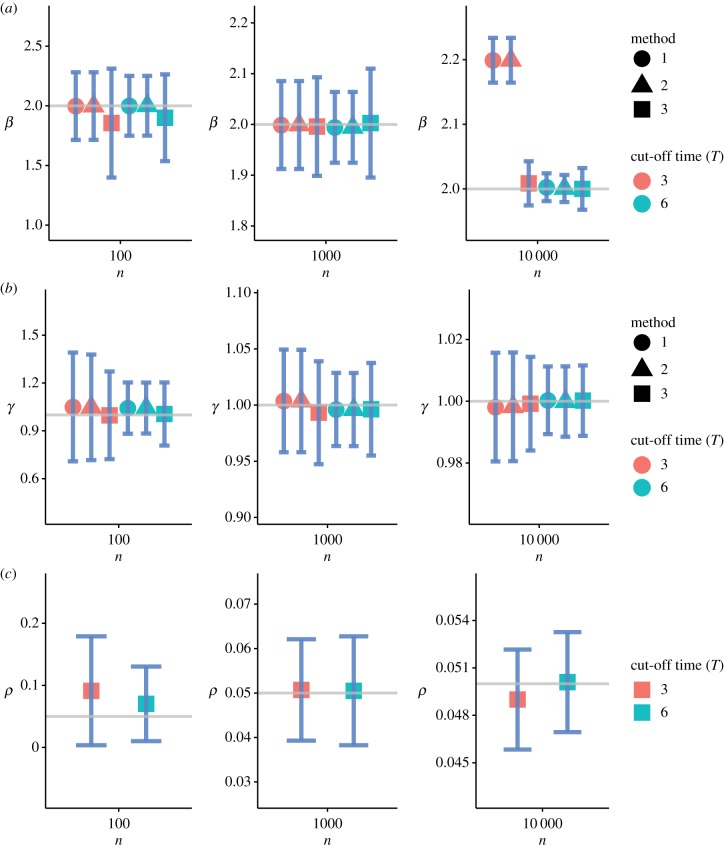
Inference under the parameter setting *θ*_2_. (*a*–*c*) The parameters *β*, *γ* and *ρ*, respectively. The solid grey lines correspond to the true parameter values. The error bars correspond to ±1.96 s.d. of each estimate. The parameter *ρ* is estimated by Method 3 only. (Online version in colour.)

**Figure 5.**
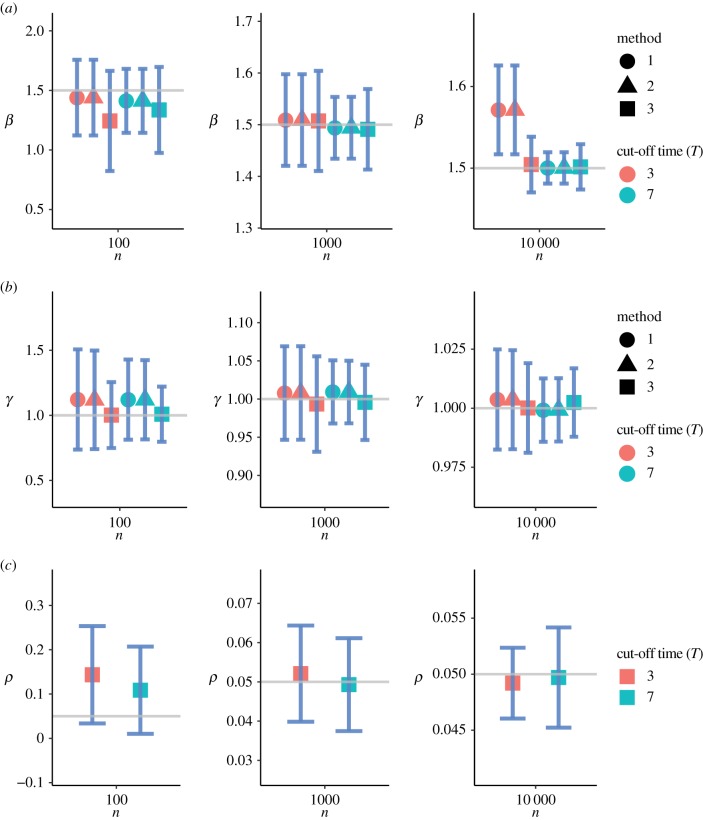
Inference under the parameter setting *θ*_3_. (*a*–*c*) The parameters *β*, *γ* and *ρ*, respectively. The solid grey lines correspond to the true parameter values. The error bars correspond to ±1.96 standard deviations of each estimate. The parameter *ρ* is estimated by Method 3 only. (Online version in colour.)

Figure 3 summarizes the numerical results of the parameter setting *θ*_1_. Figure 4 shows the results of the parameter setting *θ*_2_, and figure 5 shows the results of the parameter setting *θ*_3_. In addition to the parameter estimates (posterior means), error bars (1.96 s.d.) are also provided. These figures show that Method 3 based on the SDS likelihood fares well against Methods 1 and 2 based on the exact likelihood. Barring minor exceptions, Method 3 yielded accurate estimates for all three parameters *β*, *γ* and *ρ* even for relatively small values of *n*. The results for *n* = 10^2^ are particularly encouraging. Tables 2 and 3 show that the mean squared error (MSE) decreases with increasing *n* across all three methods. As expected, the quality of inferences for the large cut-off time settings is better than that for the small cut-off time settings.

**Table 2.** Summary of the numerical results for the longer cut-off times. Here, the values of *T* are 9 for *θ*_1_, 6 for *θ*_2_ and 7 for *θ*_3_ such that *T* is near the end of the epidemic process (also see figure 8). Method 3 yields accurate estimates without requiring knowledge of the size of the susceptible population *n*. Values in italics indicate the results corresponding to the best performing method.

			*β*			*γ*			*ρ*
	*n*	statistics	Method 1	Method 2	Method 3	Method 1	Method 2	Method 3	Method 3
*β* = 2	10^4^	Avg.	2.0067	2.0067	*2.0032*	*0.5002*	0.5002	0.4980	*0.0492*
		(MSE)	(0.00046)	(0.00046)	(0.00082)	(0.00002)	(0.00002)	(0.00004)	(0.00001)
*γ* = 0.5	10^3^	Avg.	*2.0033*	2.0033	1.9868	0.4996	*0.4997*	0.4974	*0.0521*
		(MSE)	(0.00334)	(0.00334)	(0.00883)	(0.00024)	(0.00024)	(0.00028)	(0.00014)
*ρ* = 0.05	10^2^	Avg.	2.0433	*2.0432*	1.8890	0.5055	0.5055	*0.4950*	*0.0636*
		(MSE)	(0.04238)	(0.04236)	(0.07655)	(0.07502)	(0.00284)	(0.00230)	(0.00178)
*β* = 2	10^4^	Avg.	2.0026	2.0007	*2.0000*	1.0003	*0.9999*	1.0002	*0.0501*
		(MSE)	(0.00046)	(0.00044)	(0.00101)	(0.00012)	(0.00013)	(0.00013)	(0.00001)
*γ* = 1	10^3^	Avg.	1.9942	1.9942	*2.0027*	0.9961	0.9960	*0.9963*	*0.0505*
		(MSE)	(0.00489)	(0.00489)	(0.01151)	(0.00107)	(0.00108)	(0.00172)	(0.00015)
*ρ* = 0.05	10^2^	Avg.	*2.0002*	2.0005	1.8997	1.0425	1.0431	*1.0056*	*0.0702*
		(MSE)	(0.06295)	(0.0628)	(0.14231)	(0.02772)	(0.02748)	(0.03915)	(0.00402)
*β* = 1.5	10^4^	Avg	*1.5003*	1.5003	1.5016	0.9992	*0.9993*	1.0024	*0.0497*
		(MSE)	(0.00037)	(0.00037)	(0.00078)	(0.00018)	(0.00018)	(0.00022)	(0.00002)
*γ* = 1	10^3^	Avg.	1.4940	*1.4941*	1.4911	1.0094	*1.0092*	0.9957	*0.0493*
		(MSE)	(0.00362)	(0.00362)	(0.00615)	(0.00180)	(0.00177)	(0.00245)	(0.00014)
*ρ* = 0.05	10^2^	Avg.	1.4126	*1.4127*	1.3362	1.1211	1.1199	*1.0090*	*0.1087*
		(MSE)	(0.0796)	(0.07962)	(0.15705)	(0.10955)	(0.10715)	(0.04502)	(0.01313)

**Table 3.** Summary of the numerical results for the shorter cut-off times. Here, we fix *T* = 3 so that the epidemic process is near its peak at *T* (also see figure 8). Method 3 yields accurate estimates without requiring knowledge of the size of the susceptible population *n*. Values in italics indicate the results corresponding to the best performing method.

			*β*			*γ*			*ρ*
	*n*	statistics	Method 1	Method 2	Method 3	Method 1	Method 2	Method 3	Method 3
*β* = 2	10^4^	Avg.	2.0443	2.0443	*2.0009*	0.4996	0.4996	*0.5010*	*0.0494*
		(MSE)	(0.00221)	(0.00221)	(0.00082)	(0.00006)	(0.00006)	(0.00007)	(0.00001)
*γ* = 0.5	10^3^	Avg.	2.0041	*2.0040*	2.0134	0.5034	*0.5033*	0.4956	*0.0506*
		(MSE)	(0.00545)	(5.44670)	(0.00940)	(0.00067)	(0.00067)	(0.00053)	(0.00012)
*ρ* = 0.05	10^2^	Avg.	2.0101	*2.0100*	1.8631	0.5059	0.5069	*0.5004*	*0.0700*
		(MSE)	(0.07191)	(7.19654)	(0.10753)	(0.00669)	(0.00677)	(0.00962)	(0.00240)
*β* = 2	10^4^	Avg.	2.1991	2.1991	*2.0086*	0.9981	0.9982	*0.9992*	*0.0490*
		(MSE)	(0.04083)	(0.04083)	(0.00124)	(0.00031)	(0.00031)	(0.00023)	(0.00002)
*γ* = 1	10^3^	Avg.	*1.9989*	1.9989	1.9958	1.0037	*1.0036*	0.9932	*0.0507*
		(MSE)	(0.00751)	(0.00751)	(0.00945)	(0.00210)	(0.00210)	(0.00214)	(0.00013)
*ρ* = 0.05	10^2^	Avg.	1.9979	*1.9980*	1.8553	1.0499	1.0474	*0.9973*	*0.0912*
		(MSE)	(0.08047)	(0.08043)	(0.22925)	(0.11915)	(0.11203)	(0.07570)	(0.00939)
*β* = 1.5	10^4^	Avg	1.5713	1.5713	*1.5044*	1.0037	1.0036	*1.0001*	*0.0492*
		(MSE)	(0.00804)	(0.00804)	(0.00118)	(0.00046)	(0.00046)	(0.00036)	(0.00001)
*γ* = 1	10^3^	Avg.	1.5091	1.5091	*1.5073*	1.0079	1.0080	*0.9935*	*0.0521*
		(MSE)	(0.00794)	(0.00794)	(0.00945)	(0.00381)	(0.00381)	(0.00396)	(0.00016)
*ρ* = 0.05	10^2^	Avg.	*1.4398*	1.4398	1.2439	1.1220	1.1192	*1.0020*	*0.1435*
		(MSE)	(0.10451)	(0.10461)	(0.24216)	(0.16303)	(0.15773)	0.06412)	(0.02082)

Since *ρ* is assumed known for Methods 1 and 2, it is estimated only in Method 3. Figures 3*c*, 4*c* and 5*c* show that the quality of estimation is sometimes poor when *n* is small. Note the *n* = 10^2^ case in particular. Nevertheless, it is estimated accurately when *n* is moderately large.

Further numerical results and explanations are provided in appendix C. Method 3 seems to have a slightly larger variance than the other two methods. Even though visual inspection suggests that Method 3 achieves comparable performance against Method 2 and Method 3, a more objective criterion would be useful. Such a criterion should take into account both the biases and the MSE of the methods. For instance, information criteria such as the focused information criterion [37] can be used for this purpose. However, our intention here is not to find which method performs the best, but rather to find how the approximate SDS likelihood performs against the exact likelihoods. Since figures 3–5 and the additional results in appendix C provide satisfactory evidence in favour of Method 3 and give adequate insight into its performance, we do not perform any further comparative analysis. Instead, we apply the SDS likelihood to a real dataset in the next section.

## 4. Data analysis

In the autumn of 2009, a new strain of influenza spread around the world after its initial outbreak in the state of Veracruz, Mexico in April 2009. The influenza A(H1N1)pdm09 virus was a triple reassortment of bird, swine and human flu viruses further combined with a Eurasian pig influenza virus [38]. Unlike most strains of influenza, this influenza A(H1N1) virus did not disproportionately infect adults older than 60 years, and it spread easily among young, healthy adults. This feature of the virus resulted in multiple outbreaks of the disease on college campuses across the continental USA. An outbreak on the campus of Washington State University (WSU) in Pullman, Washington began in late August 2009, upon the return of students for the autumn semester. Over a period of slightly more than three months, almost 2300 students were seen at the campus health centre with influenza-like illnesses that were treated as influenza A(H1N1) infections.^2^

**Figure 6.**
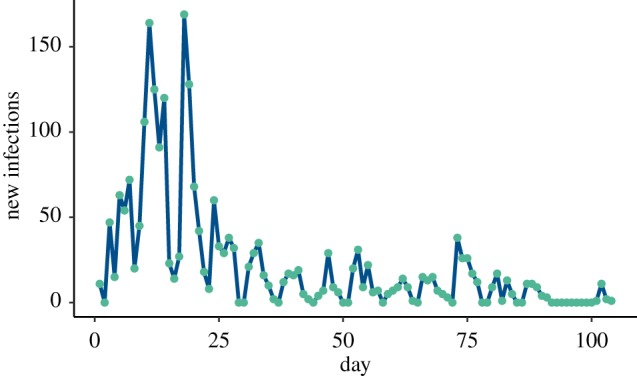
Daily new infection counts from WSU H1N1 outbreak. (Online version in colour.)

Figure 6 shows daily counts of new infections for 105 days beginning on 22 August 2009. The counts were obtained directly from the cases of ‘influenza-like illness’ among students who visited or called the WSU Student Health Services seeking care. In our statistical analysis, the collected daily counts were considered as records of ‘new infectives’. This particular dataset is interesting because it was obtained from an approximately closed population. The WSU campus is located in a town with a large student population (around 18 000 students) and a relatively small resident population (around 9000 residents). The location is relatively remote, with an average population density of only eight households per square mile in the surrounding rural areas.

As discussed in an earlier analysis of this dataset [38], these data may have been subject to both over-reporting and under-reporting: Some students may have assumed they had H1N1 when they had other influenza-like illnesses, while some students infected with H1N1 may not have sought medical care. However, such misreporting was considered to be relatively minor compared to the overall counts in the dataset [39]. This dataset was analysed earlier using a stochastic SIR model with parameters estimated using both likelihood-based and least-squares methods. Here, we re-analyse it using the SDS likelihood, emphasizing its multilevel nature by showing how the shape of the epidemic curve reflects changes in risk of infection in students who were susceptible.

The density of the infection time (conditional on *T*_*I*_ < ∞) is given by $f_{\tau }(t)=-{\dot{s}}_{t}/\tau$ (see equation (2.5)). Consequently, for the collection of *n* individuals at risk out of which *k* are seen to be infected at times *t*_1_ < ⋯ < *t*_*k*_ < *T* where *T* < ∞ in the observation time horizon (i.e. censoring time), we have the log-likelihood function for infection times

$$\ell_{I}(t_{1},\ldots ,t_{k}|\theta ,n)=(n-k)\log s_{T}+\sum_{i=1}^{k}\log f_{\tau }(t_{i}),$$

where *θ* = (*β*, *γ*, *ρ*) is the vector of free parameters, with *τ* being an implicit function of *θ* according to equation (2.3). Note that the above likelihood is conditional on the number of individuals at risk *n*, which is also typically unknown, and that the value 0 ≤ *k* ≤ *n* is a random variable. In particular, if *T* is sufficiently large, we have approximately k∼BINOMIAL(n,τ). Note that this implies in particular that if we do not know the value of *n* but have observed *k*, a reasonable estimate of the former is *k*/*τ*. In general, to impute a value of *n*, we could take n∼NEGBINOM(k,τ), the negative binomial distribution. Conditionally on the value of *k* the (unobserved) recovery likelihood is then the usual log likelihood for the exponential survival model. Assuming *r* individuals have recovered after infectious periods *w*_1_ < · · · < *w*_*r*_ < *T*, we have

$$\ell_{R}(w_{1},\ldots ,w_{r}|\theta ,k)=(k-r)\log H_{\gamma }(T)+\sum_{i=1}^{r}\log h_{\gamma }(w_{i}),$$

where $H_{\gamma }(\cdot )$ and $h_{\gamma }(\cdot )$ are, respectively, the survival function and the probability density function of the exponential distribution with rate *γ*. Averaging the infectious periods used in the previous analysis [38,39], we assume here that the recovery times have an exponential distribution with mean *γ*^−1^ = 5.5 days (see also [40,41]), so *γ* was not estimated. The complete log-likelihood conditional on the population size *n*, the parameters and observables is then

$$\begin{array}{c} \ell_{0}(t_{1}\ldots ,t_{k},w_{1},\ldots ,w_{r}|\theta ,n)=\ell_{I}(t_{1},\ldots ,t_{k}|\theta ,n)\\ \;\;\;\;+\ell_{R}(w_{1},\ldots ,w_{r}|\theta ,k). \end{array}$$

Based on this SDS likelihood, algorithm 4.1 may be used for obtaining the posterior distributions of the parameters *θ* and *n* given the WSU dataset.


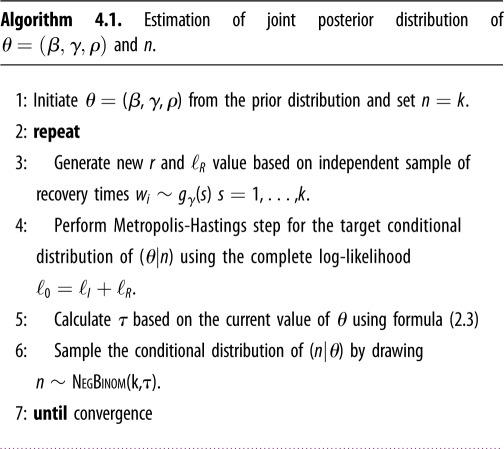


**Figure 7.**
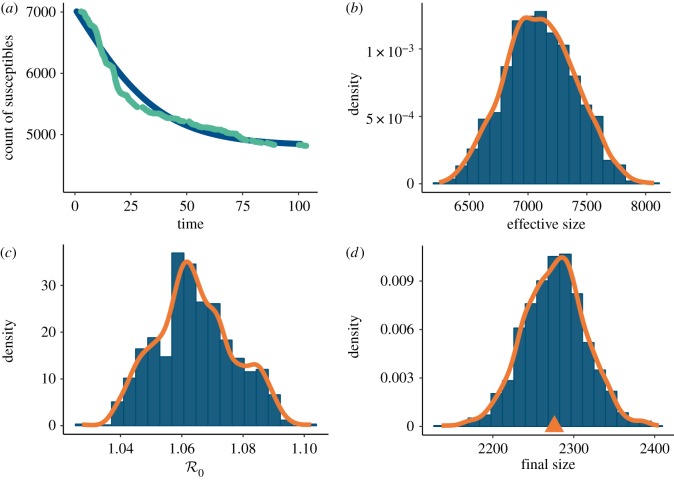
(*a*) Fitted (blue) versus observed (green) *s*_*t*_ curves and (*b*) the posterior distribution of the effective population size (*n*). Both curves are conditional on the effective population size. Posterior distributions of (*c*) the basic reproduction number $R_{0}$ and (*d*) the final epidemic size. The distribution of the latter may be used to validate the model against actually observed data. The mark is placed at the actually observed epidemic size of 2276.

**Figure 8.**
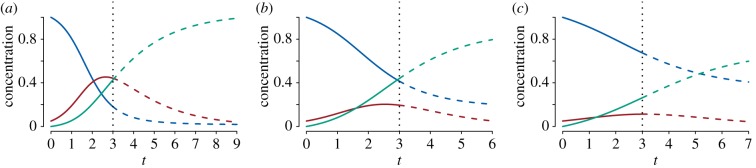
The SIR curves for the three different parameter values considered in §3.4. The initial values are *S*_0_ = 1, *R*_0_ = *ρ* and *R*_0_ = 0. The vertical dotted lines represent the cut-off times. (*a*) *β* = 2, *γ* = 0.5, *ρ* = 0.05, (*b*) *β* = 2, *γ* = 1.0, *ρ* = 0.05, (*c*) *β* = 1.5, *γ* = 1.0, *ρ* = 0.05. (Online version in colour.)

The results of applying algorithm 4.1 to the WSU dataset are summarized in table 4 and in figure 7. As in previous sections, independent, non-informative gamma priors were used for *θ*. The uniform (improper) prior was used for *n*. The maximum *a posteriori* estimate (MAP) of the effective population size (population at risk) was found to be *n* = 7051. This is much smaller than the value of approximately 18 000 (total WSU student body) assumed in the previous analyses [38,39]. Consequently, the MAP value of $R_{0}\approx 1.06$ is slightly smaller than that obtained in the previous analysis, and the SDS-based MAP for *ρ* is substantially larger than other estimates of the initially infected. Contrary to previous analysis [39], these values suggest that the high peak of an epidemic in early days of the academic year was not caused by high infectivity among newly infected students but rather by a high number of already infected individuals (high value of *ρ*). This point was already made in [38].

**Table 4.** The values of posterior parameter estimates and their credibility bounds based on the hybrid Gibbs sampler given the WSU data in figure 6.

parameter	MAP	90% credibility
*n*	7051	(6602, 7581)
*β*	0.1887	(0.185, 0.196)
*ρ*	0.0423	(0.04, 0.045)
$R_{0}$	1.06	(1.04, 1.09)

## 5. Discussion

In this paper, we present a new way of using classical SIR-type epidemic models for statistical inference. Our method addresses all four problems identified in §1. Indeed, parameter estimation based on the SDS likelihood (described in §3) does not require the effective population size *n*, addressing problem 1. The SDS likelihood, being a direct consequence of the SDS interpretation of the SIR equation (1.1), provides a principled way of specifying a likelihood function from epidemiological field data where the effective population size is unknown but large, addressing problem 2. Although we do not explicitly illustrate this here, the independence of individuals’ contributions to the SDS likelihood also addresses the problem of aggregation over individuals (problem 3) and over time (problem 4). Moreover, due to its product form, the SDS likelihood method is easier to implement and analyse than methods based on partially observed CTMC (e.g. the Doob–Gillespie likelihood).

The SDS method allows a novel approach to the monitoring of epidemics. Instead of longitudinally counting the number of infections, a random sample of individuals can be monitored continuously for changes in their health status. This is akin to a sentinel sensor network. Similar ideas have been routinely explored in communication networks literature in computer science (e.g. network probing and monitoring) [42]. The use of individual-level longitudinal data rather than counts allows much greater flexibility in estimating the effects of covariates (e.g. vaccination status) on infectiousness and susceptibility, and it extends easily to non-Markov transmission models.

Using the SDS likelihood, it typically suffices to have much smaller sample of transition data than other inference methods such as the exact likelihood method. Due to the asymptotic independence of infection and recovery times of individuals (see §2), the SDS likelihood takes a particularly simple form, facilitating a convenient implementation of a suitable MCMC scheme. We have made our code implementation of the SDS likelihood and MCMC scheme publicly available [43].

The SDS framework proposed here can be readily extended to accommodate a wide class of compartmental models with some partial ordering among compartments. The classical SIR model has been chosen here as an important example to illustrate the ideas underpinning SDS likelihoods. Indeed, the machinery developed in the present paper goes beyond compartmental SIR models, and it can be applied to more general epidemic processes as well as to many compartmental models arising in physics and chemistry. In particular, we believe SDS likelihoods can be applied to certain subclasses of chemical reaction network models in which the individual species molecules can be tracked as they undergo chemical reactions.

In many studies of epidemiological field data, the effective population size is assumed to be very large. For instance, a total population size of 10^6^ was assumed in [44,45]. Our method is particularly appropriate for such settings. For smaller populations, knowledge of the rate of convergence of the scaled processes to the LLN limit is crucial for assessing the quality of inference based on the SDS likelihood. Therefore, to fully evaluate the appropriateness of the SDS approximation, one should first establish an LDP for the scaled process of interest. This is particularly important for small-scale epidemics. Even though our numerical results are encouraging for values of *n* as small as 100, quantifying the rate of convergence will be useful. Although we did not consider an LDP in this paper, we believe that the standard techniques [21–23,25,26,46] can be applied for this purpose in our context.

Another direction of future investigation will be to consider network-based systems and non-Markovian systems. For many epidemiological scenarios, the mass-action assumption is untenable. Several network-based models have been proposed in recent times [47–49]. Asymptotic study of those models in the form of various large-graph limits has also been done [50–52]. Therefore, extending our method to network-based models appears to be a natural next step.

## Figures and Tables

**Figure 9.**
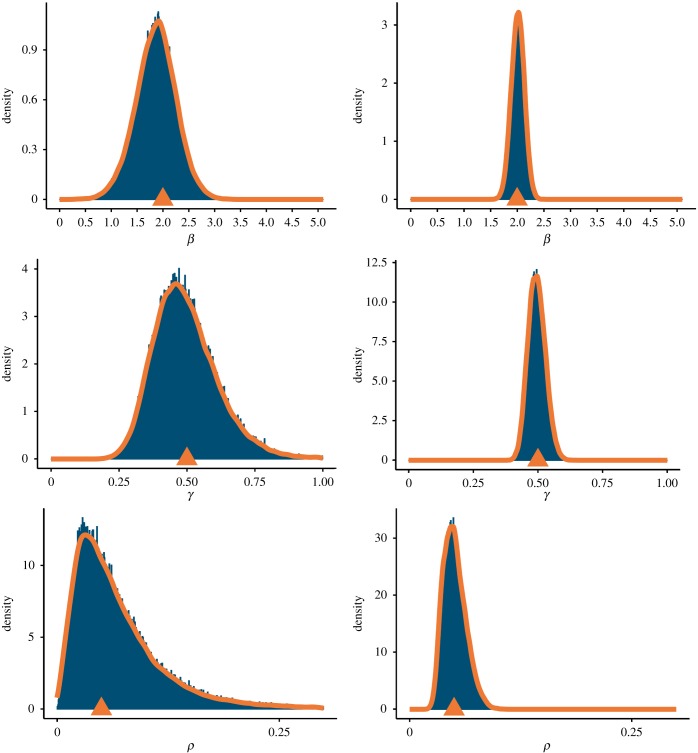
The posterior distributions of the Method 3 estimators of *β*, *γ* and *ρ* based on the SDS likelihood for the smaller cut-off time (*T* = 3). The left-hand panels correspond to *n* = 10^2^, and the right-hand panels correspond to *n* = 10^3^. The true parameter values are *β* = 2, *γ* = 0.5 and *ρ* = 0.05 (parameter setting *θ*_1_). (Online version in colour.)

**Figure 10.**
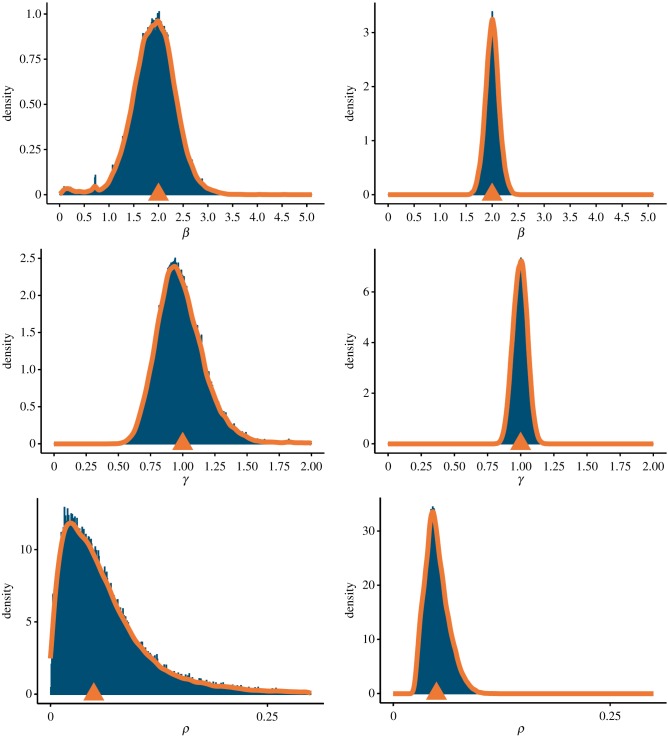
The posterior distributions of the Method 3 estimators of *β*, *γ* and *ρ* based on the SDS likelihood for the larger cut-off time (*T* = 6). The left-hand panels correspond to *n* = 10^2^, and the right-hand panels correspond to *n* = 10^3^. The true parameter values are *β* = 2, *γ* = 1 and *ρ* = 0.05 (parameter setting *θ*_2_). (Online version in colour.)

**Figure 11.**
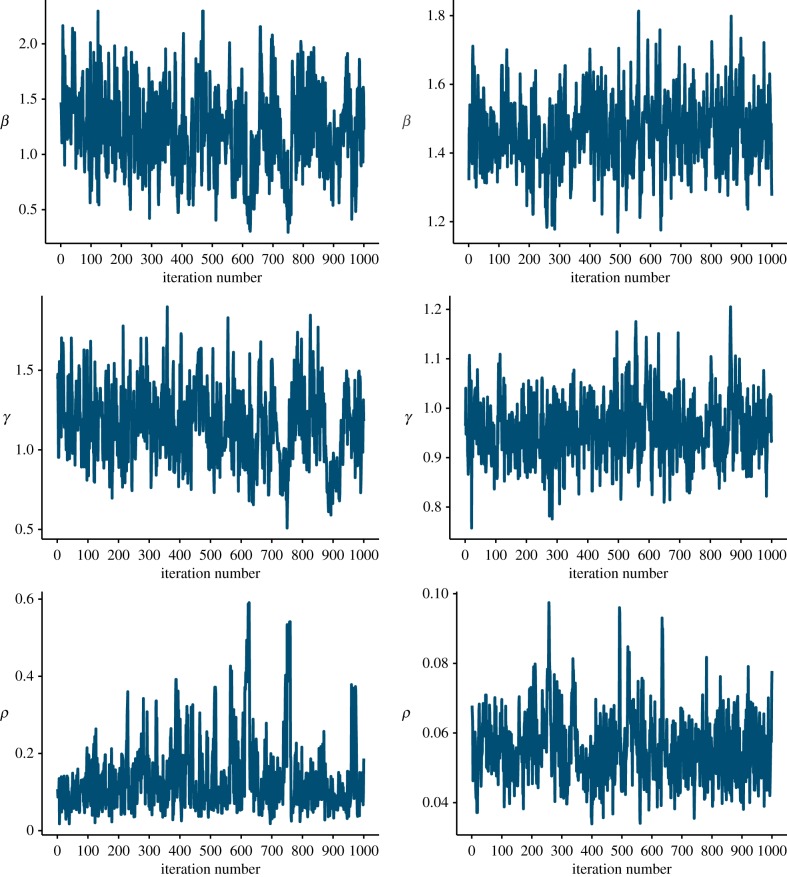
The thinned trace of a single Markov chain in the MCMC implementation of Method 3 for the smaller cut-off time (*T* = 3). Separate panels are shown for each of the parameters *β*, *γ*, and *ρ*. The left-hand panels correspond to *n* = 10^2^, and the right-hand panels correspond to *n* = 10^3^. The true parameter values are *β* = 1.5, *γ* = 1 and *ρ* = 0.05 (parameter setting *θ*_3_). (Online version in colour.)

## Endnotes

1 A random variable is said to be interval-censored when it cannot be observed exactly and is only known to lie within an interval.

2 In fact, as described in [39], for the first 10 days of the outbreak, all suspected cases were tested and laboratory-confirmed to be H1N1, after which all cases were considered H1N1.

## Data accessibility

This article has no additional data.

## Appendix A. Mathematical background

**A.1. Lumpability of a Markov chain**

Consider a CTMC *C*_*t*_ on a state space Y:={1,2,…,K} for a finite positive integer *K*. Given a partition $\left\{Y_{1},Y_{2},\ldots ,Y_{M}\right\}$ of Y, define the process ${\tilde{C}}_{t}$ such that ${\tilde{C}}_{t}:=i$ whenever $C_{t}\in Y_{i}$, for *i* = 1, 2, …, *M*. The original CTMC *C*_*t*_ is said to be strongly lumpable with respect to the partition $\left\{Y_{1},Y_{2},\ldots ,Y_{M}\right\}$ of Y if the process ${\tilde{C}}_{t}$ is also a CTMC for every choice of initial distribution of *C*_*t*_. The process ${\tilde{C}}_{t}$ is often called the aggregated or the lumped process. Intuitively, lumpability is the property that disjoint sets of states can be identified by representative states such that the induced stochastic process on the representative states (which we call the aggregated or lumped process) is also Markovian for every choice of initial distribution of the original CTMC. In our individual-level model described in §1.1, the representative states are given by the partition $\left\{X_{1},X_{2},\ldots ,X_{L}\right\}$ of the state space $X^{n+m}$. The representative states then correspond to the population counts *S*(*t*), *I*(*t*), *R*(*t*).

The (strong) lumpability^3^ of a CTMC can also be described in terms of lumpability of a linear system of ODEs. Consider the linear system $\dot{y}=yA$, where *A* = ((*a*_*i*,*j*_)) is a *K* × *K* matrix (representing the transition rate or the infinitesimal generator matrix of the corresponding CTMC on state space Y).

Definition A.1 (lumpability of a linear system [10,18]). The linear system $\dot{y}=yA$ is said to be lumpable with respect to a partition $\left\{Y_{1},Y_{2},\ldots ,Y_{M}\right\}$ of Y, if there exists an *M* × *M* matrix *B* = ((*b*_*i*,*j*_)) satisfying Dynkin’s criterion (i.e. if $b_{i,j}=\sum_{l\in Y_{\;j}}a_{u,l}=\sum_{l\in Y_{\;j}}a_{v,l}$ for all $u,v\in Y_{i}$). The matrix *B* is often called a lumping of *A*. The following is immediate: if *B* is a lumping of *A*, then there exists an *K* × *M* matrix *V* such that *AV* = *VB*.

Refer to [16,17,53] for further reading and numerous characterizations of Markov chain lumpability.

## Appendix B. Additional pseudocode

For the sake of completeness, we provide some additional pseudocode for implementing popular statistical procedures. The first pseudocode is for simulating trajectories of a CTMC following the well-known Doob–Gillespie algorithm.

The MCMC procedure for drawing posterior samples using the RAM algorithm can be implemented by the following pseudocode.

## Appendix C. Additional numerical results

Here, we provide additional numerical results. In particular, we show the posterior plots and crucial diagnostic statistics for the MCMC methods.

The cut-off times are chosen based on figure 8. The idea is to study the impact of censoring on the quality of inference. For each parameter setting, we chose two cut-off times: one near the peak of the epidemic and one near the end of the epidemic. The vertical lines in figure 8 indicate the smaller cut-off time for each of the three settings of the parameter values.

Our numerical results are summarized in tables 2 and 3. For the longer cut-off times, table 2 provides a summary of the simulation results for the three parameter settings and different initial numbers of susceptibles *n*. Here, the values of *T* are 9 for *θ*_1_, 6 for *θ*_2_ and 7 for *θ*_3_. In each case, the epidemic is almost at its end by time *T* (figure 8). The first three columns show estimates of *β* from Methods 1, 2 and 3. Similarly, the next three columns show estimates of *γ*. The last column shows Method 3 estimates of *ρ* (recall that *ρ* is known exactly for Method 1 and Method 2). The rows of the table are divided into three parts corresponding the parameter settings *θ*_1_, *θ*_2_ and *θ*_3_. Each of the three parts is further subdivided into the three different susceptible population sizes *n* = 10^2^, 10^3^ and 10^3^. Finally, in each cell, we show the average of 100 posterior means and the MSE of parameter estimators. As we can see, Method 3 based on the SDS likelihood yields accurate estimates for all three parameters *β*, *γ* and *ρ* even for relatively small values of *n* (see the results for *n* = 10^2^).

Whereas table 2 considers data collected until a *T* near the end of an epidemic, table 3 considers data with cut-off *T* = 3 that is close to the peak of an epidemic. (See figure 8 for a visualization of the SIR curves corresponding to these three parameter settings truncated at *T* = 3 by a vertical line.) The table formats are identical. Since the inference is based on heavily truncated data, the MSE in table 3 are higher than those in table 2. Also, the sharp decrease in MSE with increasing *n* in table 2 is less pronounced in table 3. Nevertheless, the estimates obtained are still quite accurate. Also, the MSE for Method 3 are slightly better than those of Method 1 or 2. Interestingly, the parameter *ρ* is accurately estimated by Method 3.

In figures 9 and 10, we show the posterior distributions of the Method 3 estimators of *β*, *γ* and *β* based on the SDS likelihood. To avoid repetition, we show only two posterior plots: figure 9 shows results for the parameter setting *θ*_1_ under the smaller cut-off time, and figure 10 shows results for the parameter setting *θ*_2_ under the larger cut-off time. As shown in tables 3 and 2, the variances of the posterior distributions shrink drastically as we increase *n* from 10^2^ to 10^3^. We do not show the posterior distributions for the *n* = 10^4^ case because it does not provide any additional insights into the quality of the inference procedure except for the fact that the posterior variance further reduces.

Finally, figure 11 shows additional diagnostic statistics for the MCMC implementation of Method 3. We show the thinned trace of a single Markov chain for *n* = 10^2^ and 10^3^. As expected, the chain mixes faster when *n* = 10^3^ than when *n* = 10^2^ because Method 3 is based on an LLN of the scaled Poisson processes keeping track of the population counts. As before, we omit the *n* = 10^4^ case. For completeness, we consider the third parameter setting *θ*_3_ in figure 11. The Markov chains also converge for the other parameter settings (not shown).
